# Carbohydrate metabolism and cytology of S-type cytoplasmic male sterility in wheat

**DOI:** 10.3389/fpls.2023.1255670

**Published:** 2023-10-16

**Authors:** Shijie Ge, Fugong Ding, Bimpong Daniel, Cuicui Wu, Mingyang Ran, Chi Ma, Yuhang Xue, Die Zhao, Yike Liu, Zhanwang Zhu, Zhengwu Fang, Gaisheng Zhang, Yingxin Zhang, Shuping Wang

**Affiliations:** ^1^ Ministry of Agriculture and Rural Affairs (MARA) Key Laboratory of Sustainable Crop Production in the Middle Reaches of the Yangtze River/College of Agriculture, Yangtze University, Jingzhou, Hubei, China; ^2^ Food Crops Institute, Hubei Academy of Agricultural Sciences, Wuhan, Hubei, China; ^3^ College of Agriculture, Northwest Agricuture and Forestry (A&F) University, Yangling, Shaanxi, China

**Keywords:** cytoplasmic male sterility, cytomorphology, chloroplast proteomics, carbohydrate metabolism, wheat (*Triticum aestivum* L.)

## Abstract

**Introduction:**

Cytoplasmic male sterility (CMS) is an important tool for hybrid heterosis utilization. However, the underlying mechanisms still need to be discovered. An adequate supply of nutrients is necessary for anther development; pollen abortion would occur if the metabolism of carbohydrates were hampered.

**Methods:**

In order to better understand the relationship between carbohydrate metabolism disorder and pollen abortion in S-CMS wheat, the submicroscopic structure of wheat anthers was observed using light microscopy and transmission electron microscopy; chloroplast proteome changes were explored by comparative proteomic analysis; sugar measuring and enzyme assays were performed; and the expression patterns of carbohydrate metabolism-related genes were studied using quantitative real-time PCR (qRT-PCR) method.

**Results:**

These results indicated that the anther and microspore in S-CMS wheat underwent serious structural damage, including premature tapetum degeneration, nutritional shortage, pollen wall defects, and pollen grain malformations. Furthermore, the number of chloroplasts in the anthers of S-CMS lines decreased significantly, causing abnormal carbohydrate metabolism, and disintegration of osmiophilic granules and thylakoids. Meanwhile, some proteins participating in the Calvin cycle and carbohydrate metabolism were abnormally expressed in the chloroplasts of the S-CMS lines, which might lead to chloroplast dysfunction. Additionally, several key enzymes and genes related to carbohydrate metabolism were significantly inhibited in S-CMS.

**Discussion:**

Based on these results, we proposed a carbohydrate metabolism pathway for anther abortion in S-type cytoplasmic male sterility, which would encourage further exploration of the pollen abortion mechanisms for CMS wheat.

## Introduction

Male sterility is widespread in higher plants, which plays an important role in crop breeding, and is also one of the main agronomic traits for studying the utilization of crop heterosis (Liu et al., 2018a; Wang et al., 2018). Until now, CMS has been characterized in more than 140 natural species (Wang et al., 2019). Wheat is one of the most widely planted crop in the world, and the use of CMS provides important materials and ideas for wheat heterosis, and is considered as one of the most effective ways to improve wheat yield and quality (Singh et al., 2015).

CMS is defective in producing functional pollen, where pollen abortion happened at various developmental stages (Zhang et al., 2022). Previous studies have suggested that 38 species exhibited pollen abortion during anther development, 15.5% of them happened at the early stages of meiosis (13.5% in dicotyledonous plants, 2% in monocotyledonous plants), 57.5% happened at the tetrad stage (29% in dicotyledonous plants, 28.5% in monocotyledonous plants), and 27% happened at the microspore developmental stage (7.5% in dicotyledonous plants, 19.5% in monocotyledonous plants; Wang et al., 2015; Liu et al., 2018b). Based on morphological studies, the development of wheat anthers could be divided into five stages: the tetrad stage, the early uninucleate stage, the later uninucleate stage, the binucleate stage, and the trinucleate stage; and there are four different types of pollen abortion: pollen-free, uninucleate abortive, binucleate abortive and trinucleate abortive (Wang et al., 2015). Until now, more than 130 nuclear and cytoplasmic hybrids have been obtained, including K (*Aegilops kotschyi*), T (*Triticum timopheevii*), D^2^ (*Aegilops juvenalis*), Mu (*Aegilops uniaristata*), and S (*Triticum spelta*)-type sources (Yao et al., 1998; Ikeguchi et al., 1999; Liu et al., 2018a). Moreover, for different types of male sterility, pollen abortion happened at different periods, such as K-CMS belongs to tetrad abortive (Liu et al., 2018b), D^2^-CMS belongs to late-uninucleate abortive (Liu et al., 2018b), and Mu-CMS belongs to binucleate uninucleate abortive (Liu et al., 2018a). Numerous studies have revealed that the development of microspores depends on the timely degradation of tapetum cells, and early or delayed degradation of tapetum cells may directly or indirectly lead to pollen abortion, resulting in male sterility (Wang et al., 2018; Cai et al., 2021). The abortion period and tapetum developmental characteristics of male sterility in S-type wheat, however, remain unknown.

Carbohydrates are essential energy sources for pollen development (Zhu et al., 2015; Yang et al., 2017a). Sugar metabolism plays a central role in the entire biological metabolism and is closely related to protein, lipid, nucleic acid, and secondary metabolism (Rolland and Sheen, 2005; Ruan, 2014). In addition, they play a similar function as plant hormones, acting as first messengers in signal transduction pathways of gene expression regulation, assimilation and distribution, cell division and differentiation, and metabolism (Rolland and Sheen, 2005; Smeekens and Hellmann, 2014). According to studies, when higher plants reach sexual reproduction stage, a significant amount of photosynthetic products are transported to the anthers to support their normal development and the production of functional pollen; the anther also serves as an important metabolic reservoir (Rolland et al., 2006; Castro and Clément, 2007). Especially in the early stage of pollen development, the anthers are characterized by active metabolism, vigorous division and growth, and are tissues with highest pool strength in the bud of a flower (Castro and Clément, 2007). Additionally, a large amount of sugar is transported into anthers to support their normal development (Clément and Audran, 1999). In order to support the maturation of microspores into mature pollen grains, carbohydrates need to be transported into reproductive organs (Clément and Audran, 1999; Castro and Clément, 2007; Yang et al., 2017a). Disturbing sugar metabolism in anther can lead to sugar deficiency during microspore development, which can impair the pollen development and eventually result in male sterility (Dorion and Saini, 1996; Goetz et al., 2001; Oliver et al., 2010; Zhu et al., 2015; Yang et al., 2017a). Exploring the relationship between carbohydrate metabolism and anther abortion will thus help to reveal the sterility mechanism of wheat S-CMS lines.

The aim of this study was to provide insight into the cytological dynamics and carbohydrate metabolism changes of anthers in wheat S-CMS lines. Towards this objective, a comprehensive morphological and structural analysis of the anther and chloroplasts in which were conducted to study the structure variation of wheat S-CMS lines, a systematic study of the chloroplast proteome was performed to identify proteins associated with carbohydrate metabolism inducing anther abortion, and the expression profile of genes involved in sugar transport and metabolism was examined. In the present study, a carbohydrate metabolism pathway of anther abortion in S-type cytoplasmic male sterility has been established, which would provide a positive theoretical reference for finding out the regulatory mechanisms underlying male sterility of wheat.

## Materials and methods

### Plant materials

The cytoplasmic male sterile line CMS-1376A (with *Triticum spelta* L. cytoplasm) and its maintainer line XN-1376B with the same nuclear background were used in this study. To ensure complete nucleus substitution, the sterile line was developed from stable sterile lines by backcrossing with XN1376B for more than 30 generations. The microspore developmental stages were checked as described previously (Wang et al., 2015; Wang et al., 2019), including the tetrad stage (Tds), early uninucleate stage (Euns), late uninucleate stage (Luns), binucleate stage (Bns), and trinucleate stage (Tns).

### Light microscopic observation

Anthers and pistils at different developmental stages were photographed using a Motic K400 stereomicroscope (Preiser Scientific, Louisville, KY, USA). Microspores at different developmental stages were stained with 1.5% acetocarmine. Mature pollen was determined using 2% iodine-potassium iodide (I_2_-KI).

For Ehrlich’s hematoxylin staining, anthers at different developmental stages were randomly collected and fixed in FAA (70% alcohol: 37% formaldehyde: acetic acid; 18:1:1, v/v/v), embedded in paraffin, and sectioned transversely at 6 μm. All of the sections were dewaxed, dehydrated with graded series of alcohols, transferred to distilled water, and then stained with Ehrlich’s hematoxylin solution. Samples were observed using the Nikon ECLIPSE E600 (Nikon, Tokyo, Japan).

### Transmission electron microscopy observation

Freshly collected anthers were washed once with PBS and fixed in 4% (w/v) glutaraldehyde, then embedded in LR White (medium) resin. Ultrathin sections (50 to 70 nm) were sectioned on a UC6 ultramicrotome (Leica, Wetzlar, Germany) and examined under a HT7700 transmission electron microscope (Hitachi, Tokyo, Japan).

### Chloroplast proteome analysis

Fresh florets were collected for chloroplast isolation according to the method of Yang et al. (2017b) with minor modifications. About 50 g (fresh weight) of florets were homogenized with a tissue homogenizer in 500 ml of homogenization solution (0.33 mol L^-1^ sorbitol, 0.1% (w/v) BSA, 5 mmol L^-1^ EDTA, 50 mmol L^-1^ Tris-HCl (pH 8.0), 0.1% (v/v) β-ME). After filtered with four layers of Miracloth (Calbiochem, San Diego, USA), the homogenate was centrifuged at 700*× g* for 15 min, the supernatant was collected and centrifuged at 3000*× g* for 10 min. The resulting pellet of crude organelles was carefully resuspended with 10 ml of wash buffer (50 mmol L^-1^ Tris-HCl, 25 mmol L^-1^ EDTA, pH 8.0) and loaded onto a three-step sucrose gradient, bottom to top, of 60%, 45%, and 30% in wash buffer. The gradient was then centrifuged at 30,000*× g* for 2 h. The chloroplast band at the interfaces between 60% and 45% was collected with an injection needle. Then the chloroplast fraction was diluted approximately five-fold with wash buffer and centrifuged at 3,000*× g* for 15 min. The pellet was resuspended with 500 μl of wash buffer and performed for chloroplast protein isolation.

Chloroplast protein for isoelectric focusing (IEF) was extracted with TCA-acetone precipitation method (Chen et al., 2010). The concentration of proteins was determined using the Bradford method, as described by Zuo and Lundahl (2000). 2-DE was conducted as described by Song et al. (2015). Briefly, about 200 μg of protein was loaded onto a 17-cm (pH 4-7) linear pH gradient IPG strip (Bio-Rad, Hercules, CA, USA) and subjected to electrophoresis on the IPGphor apparatus (PROTEAN IEF Cell; Bio-Rad, Hercules, CA, USA). The second electrophoretic dimension used 12% SDS-PAGE. Protein spots were visualized after silver staining. Gels were scanned using a UMAX PowerLook 2100XL scanner (UMAX, Taiwan, China), and images were scanned at 600 dpi resolution for quantitative analysis using the PDQuest 8.0.1 software (Bio-Rad, Hercules, CA, USA). Three independent biological replicates were performed for all the experiments. MALDI-TOF-TOF/MS analysis and database searching for differentially expressed proteins (DEPs) were carried out according to Wang et al. (2019).

### Sugar and starch analysis

Total sugar content in anthers at different developmental stage was measured using anthrone colorimetry method, according to Wang et al. (2018). Starch content in anthers was measured according to the method of Dorion and Saini (1996) and Wang et al. (2018).

### Enzyme assays

Wheat anthers at various developmental stages were collected and used for enzyme activity analyzation. Vacuolar invertase (VIN) activity was assayed as in Sergeeva et al. (2006). The activity of cell wall invertase (CWI) was determined according to Koonjul et al. (2005).

### qPCR assay

Total RNA in anthers at different developmental stages was extracted using Trizol Reagent Kit (Invitrogen, Carlsbad, CA, USA), and then used for first-strand cDNA synthesis with the PrimeScript™ RT reagent Kit (Takara Bio, Tokyo, Japan). qRT-PCR analyses were conducted using SYBR Premix EX Taq (Takara Bio, Tokyo, Japan) with a CFX96™ Real-Time PCR Detection System (Bio-Rad, Hercules, CA, USA), according to the manufacturer’s protocols. The specific primers and reference gene primers used for qRT-PCR are all listed in **Table S1**. The relative expression levels were calculated using the 2^-^ *^△△^* ^Ct^ analysis method (Livak and Schmittgen, 2001). All reactions were performed in triplicate on one plate and repeated three times (Biological replicates).

## Results

### Phenotypic analyses of S-CMS

**Figure 1:**
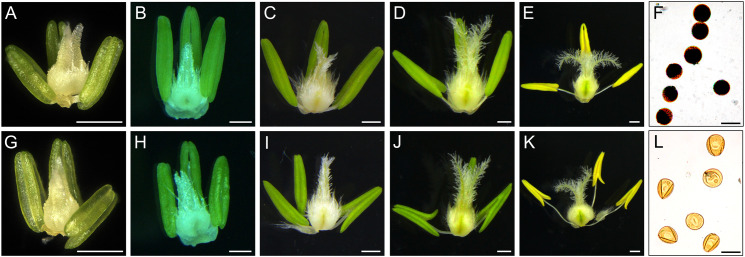
Comparison of stamen and pistil of XN1376B **(A–F)** and CMS-XN1376A **(G-L)**. **(A, G)** Tetrad stage. **(B, H)** Early uninucleate stage. **(C, I)** Later uninucleate stage. **(D, J)** Binucleate stage. **(E, K)** Trinucleate stage. **(F, L)** the 2% I_2_-KI staining pollen grains. Bars=0.5 mm **(A-E, G-K)**, 50 μm **(F, L)**.

Compared with XN1376B, the anthers of CMS-XN1376A developed normally at the tetrad stage (**Figures 1A, G**). However, from the early uninucleate stage to trinucleate stage, CMS-XN1376A anthers were slightly smaller than those of XN1376B (**Figures 1B–E, H–K**). More importantly, at the trinucleate stage, the S-CMS anther is indehiscent (**Figures 1E, K**), and its pollen could not be deeply stained with 2% I_2_-KI (**Figures 1F, L**). These findings indicated that S-CMS plants were completely pollen-sterile.

### Cytological traits of anther abortion in S-CMS

**Figure 2:**
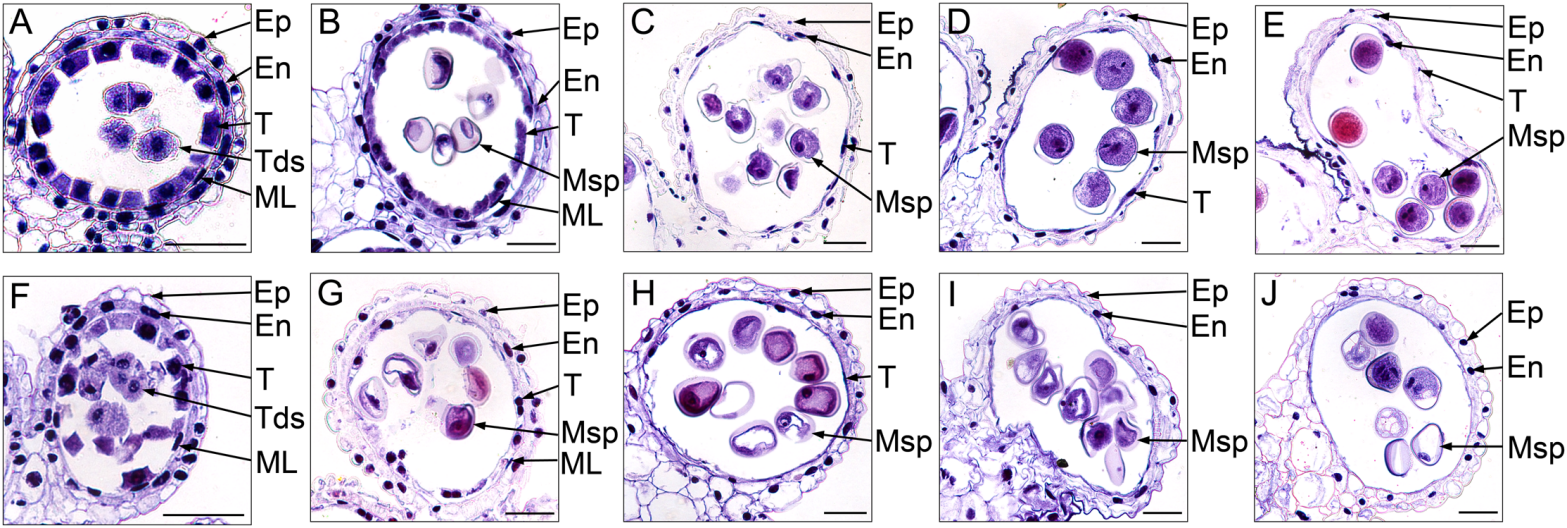
Ehrlich’s hematoxylin stained transverse sections of the anthers from XN1376B **(A–E)** and CMS-XN1376A **(F–J)**. **(A, F)** Tetrad stage. **(B, G)** Early uninucleate stage. **(C, H)** Later uninucleate stage. **(D, I)** Binucleate stage. **(E, J)** Trinucleate stage. En, endothecium; Ep, epidermis; ML, middle layer; Msp, microspore; T, tapetum; Bars= 50 μm.

To further investigate the cytological structural changes of anthers in S-CMS, we performed hematoxylin staining for the transverse section of anthers at different developmental stages (**Figure 2**). At the tetrad stage, the size of tapetal cells in CMS-XN1376A were significantly bigger than those at any other stage, and their cytoplasm could be deeply stained by Ehrlich’s hematoxylin (**Figure 2F**). In addition, there was no obvious difference between CMS-XN1376A and XN1376B. Normal epidermis, endothecium, middle layer, and tapetum were found (**Figures 2A, F**). At the early uninucleate stage, the cells of the middle layer began to disintegrate in both CMS-XN1376A and XN1376B (**Figures 2B, G**), and the tapetum cells of XN1376B remained intact (**Figure 2B**), but the cytoplasm of the CMS-XN1376A tapetum became very thin and stained lighter (**Figure 2G**). Meanwhile, the middle layers began to degenerate and became very thin in both CMS-XN1376A and XN1376B (**Figures 2B, G**). At the later uninucleate stage, the middle layers in both CMS-XN1376A and XN1376B disappeared (**Figures 2C, H**). The tapetal cells of XN1376B retained the nucleus outline, dense cytoplasm, and hill-like shape (**Figure 2C**). By contrast, the CMS-XN1376A tapetal cells became narrower and displayed discontinuously and fragmented staining patterns, which became lighter staining (**Figure 2H**). Subsequently, the tapetal cells of XN1376B remained relatively thick, with an integral narrow-band-like shape at the binucleate stage (**Figure 2D**) and a clear nucleus outline at the trinucleate stage (**Figure 2E**). However, the tapetal cells of CMS-XN1376A disintegrated into debris at the binucleate stage (**Figure 2I**) and were invisible at the trinucleate stage (**Figure 2J**). Therefore, we suggested that the premature tapetum degeneration might result in the pollen abortion of CMS-XN1376A.

**Figure 3:**
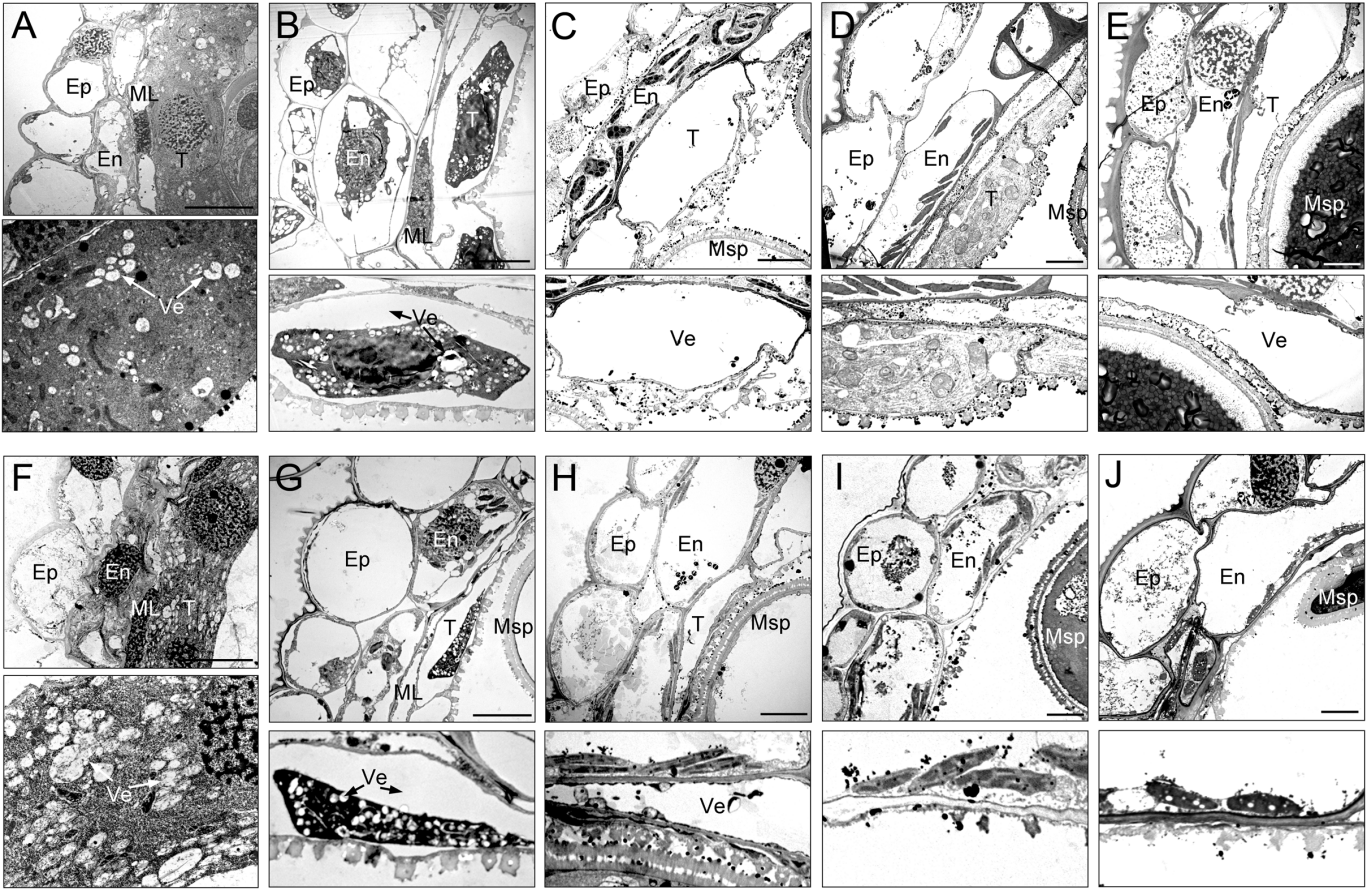
Transmission electron micrographs of the anthers from XN1376B **(A–E)** and CMS-XN1376A **(F–J)**. **(A, F)** Tetrad stage. **(B, G)** Early uninucleate stage. **(C, H)** Later uninucleate stage. **(D, I)** Binucleate stage. **(E, J)** Trinucleate stage. En, endothecium; Ep, epidermis; ML, middle layer; Msp, microspore. T, tapetum; Ve, vesicle; Bars=5 μm.

To gain a more detailed study of the abnormalities of CMS-XN1376A anthers, we observed the tapetum development of which using transmission electron microscopy (**Figure 3**). At the tetrad stage, the tapetum cells of both XN1376B and CMS-XN1376A kept intact cell outlines (**Figures 3A, F**). However, a marked degradation phenomenon was observed in the tapetum cells of CMS-XN1376A, which was manifested by decreased cell content and generation of a large number of vesicles (**Figure 3F**). Compared with XN1376B, the CMS-XN1376A tapetum cells showed significant degradation traits at the early uninucleate stage, which became relatively narrow and the volume of vesicles in them were enlarged (**Figures 3B, G**). After developed into later uninucleate stage, the cytoplasm of CMS-XN1376A tapetum cells were completely degraded, and narrow vesicles formed (**Figures 3C, H**). From binucleate stage to trinucleate stage, the tapetum remained visible in XN1376B (**Figures 3D, E**), but was completely degraded and became invisible in CMS-XN1376A (**Figures 3I, J**).

These observations indicate that the tapetum cells of CMS-XN1376A began to develop abnormally at the tetrad stage and completely degraded at the binucleate stage, which showed physiological characteristics of premature degradation.

### Microspore observations in S-CMS

**Figure 4:**
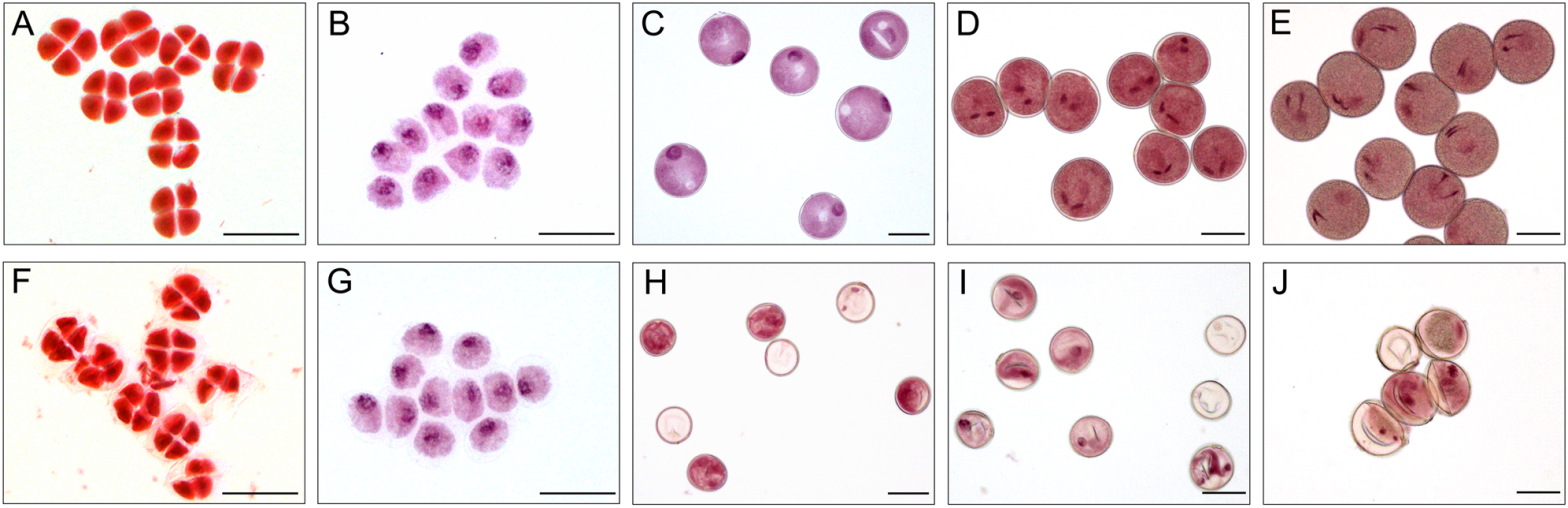
Acetocarmine stained of microspores in XN1376B **(A–E)** and CMS-XN1376A **(F-J)**. **(A, F)** Tetrad stage. **(B, G)** Early uninucleate stage. **(C, H)** Later uninucleate stage. **(D, I)** Binucleate stage. **(E, J)** Trinucleate stage. Bars=50 μm.

**Figure 5:**
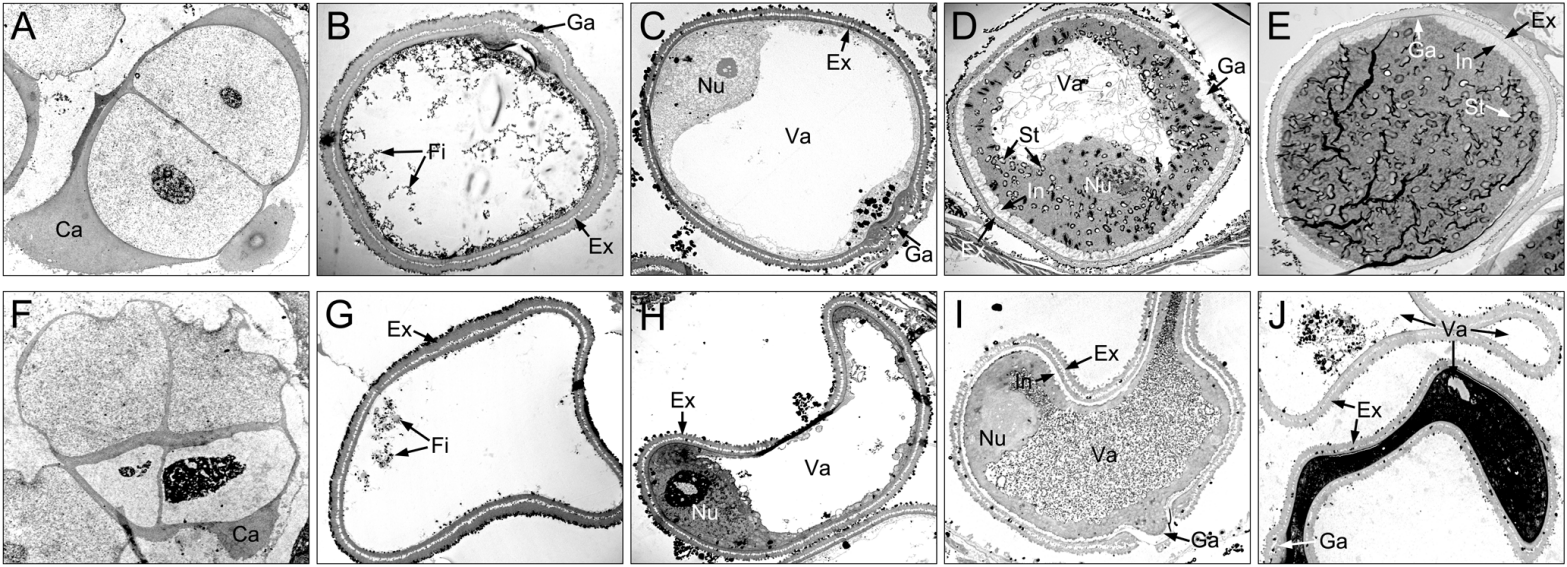
Comparison of microspores from the XN1376B **(A–E)** and CMS-XN1376A **(F–J)**. **(A, F)** Tetrad stage. **(B, G)** Early uninucleate stage. **(C, H)** Later uninucleate stage. **(D, I)** Binucleate stage. **(E, J)** Trinucleate stage. Ca, callose; Ex, exine; Fi, fibrous tissue; Ga, germination aperture; In, intine; Nu, nuclei; St, starch granules; Va, vacuole; Bars=5 μm.

The development of microspores in anthers completely depends on the tapetum cells to provide necessary nutrients and information. Once the tapetum develops abnormally, pollen abortion will inevitably happen (Wang et al., 2015; Gotelli et al., 2023). To explore the development of microspores and their relationship with the behavior of tapetum, we monitored the development of microspores using light microscopy (**Figure 4**) and transmission electron microscopy (**Figure 5**).

At the tetrad stage, there was no obvious difference in microspore cellular morphology between CMS-XN1376A and XN1376B. Tetrads of haploid microspores developed normally and could undergo meiosis to form uninucleate microspores, and a clear nucleus was observed (**Figures 4A, F**; **5A, F**). During the early uninucleate stage, microspores were released from tetrads and the nuclei were all located at the center of the cell (**Figures 4B, G**). The XN1376B microspores form a relatively thick exine, which is round in shape and contain a large amount of fibrous materials (**Figure 5B**). However, the microspore of CMS-XN1376A had a relatively thin exine, less cytoplasm, and began to show a shrinking development at this stage (**Figures 4G**; **5G**). Up to the later uninucleate stage, the microspores began to form an enormous vacuole in XN1376B and displayed an increased microspore volume; the nucleus was located opposite the germination aperture (**Figures 4C**; **5C**). In contrast, CMS-XN1376A plants produced severely irregular microspores (**Figures 4H**; **5H**). At the binucleate stage, the microspores continued to enlarge and began to accumulate nutrients, which have dense cytoplasm and two distinct nuclei (vegetative and sperm nuclei, **Figure 4D**). And as pollen began to form a relatively thick intine, a large amount of starch grains were accumulated (**Figure 5D**). In addition, the large vacuole gradually shrinks but is still clearly visible (**Figure 5D**). In contrast to XN1376B, the microspores of CMS-XN1376A are smaller and lack cytoplasm (**Figure 4I**). There is no starch granule and a large number of small vacuoles are distributed throughout the microspores (**Figure 5I**). Thus, there are some empty microspores (**Figures 4I**; **5I**). At the trinucleate stage, the mature pollen grains of XN1376B had two sperm nuclei and one vegetative nucleus, which were rich in starch, lipids, and physiologically active substances (**Figure 4E**). They had obvious exine and intine (**Figure 5E**), and could develop into mature pollen grains. However, the CMS-XN1376A microspores displayed a completely misshapen and intine disappeared pattern, and some of the microspores were empty (**Figures 4J**; **5J**).

### Morphology and proteomics analysis of chloroplast

**Figure 6:**
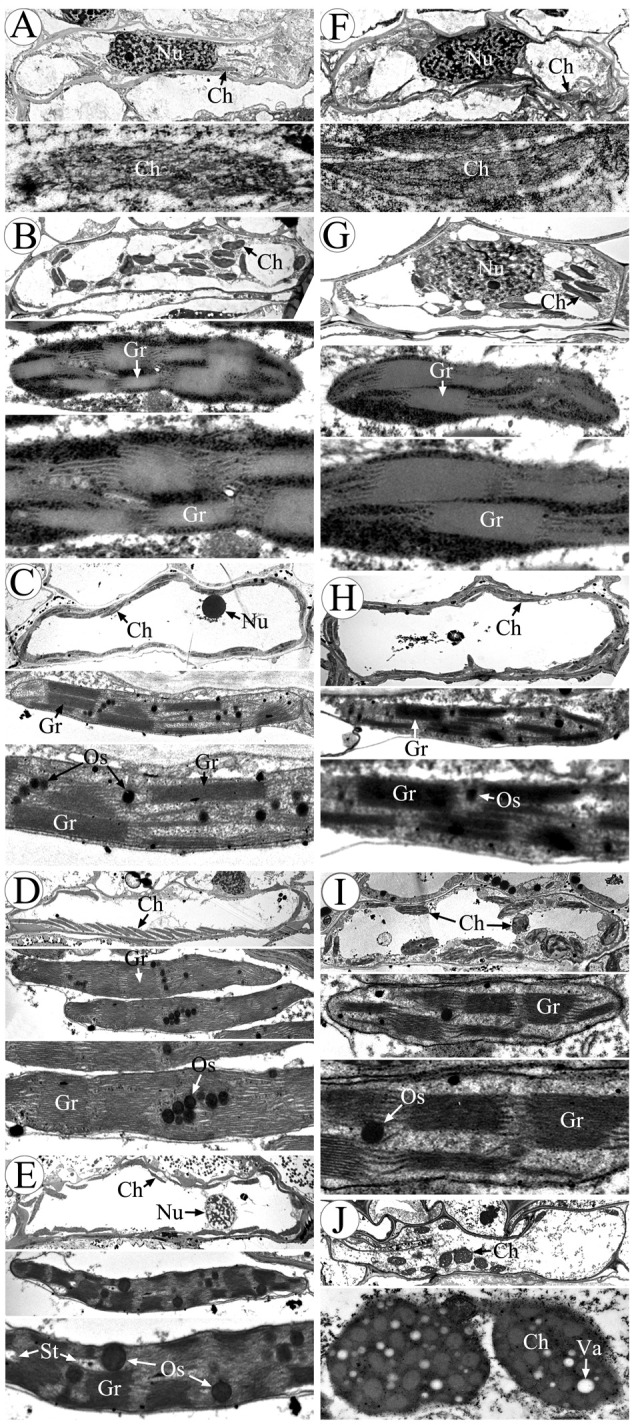
Morphology of chloroplasts in the anther by transmission electron microscopy. **(A-E)** XN1376B, **(F-J)** CMS-XN1376A, **(A, F)** Tetrad stage, **(B, G)** Early uninucleate stage, **(C, H)** Later uninucleate stage, **(D, I)** Binucleate stage, **(E, J)** Trinucleate stage, Ch, chloroplast; Gr, granum; Nu, nuclei; Os, osmiophilic granules; St, starch granules; Va, vacuole; Bars=10 μm.

Chloroplasts are organelles existing in plant cells and using sunlight, water, and carbon dioxide to produce sugar for plants (Ruan, 2014; Sabrina et al., 2022). We observed chloroplasts of the anther at various developmental stages using transmission electron microscopy to gain a more thorough understanding of the abnormalities of chloroplast ultrastructure in CMS-XN1376A (**Figure 6**). There was no grana lamella; instead, chloroplasts were only seen in the endothecium throughout the anther development and as proplastids at the tetrad stage (**Figures 6A, F**). Subsequently, these proplastids developed into chloroplasts with a complete membrane system, including normal granum and thylakoids at the early uninucleate stage, and thylakoids had a distinct lamellar structure (**Figures 6B, G**). There was no obvious difference in chloroplast morphology between CMS-XN1376A and XN1376B. At the later uninucleate stage, the chloroplasts of XN1376B had complete structure and were uniformly distributed against the cell wall. The thylakoids were superimposed on each other to form a large number of clearly visible grana. Many osmiophilic granules were accumulated and homogeneously scattered in the chloroplasts (**Figure 6C**). However, the length of chloroplasts in the CMS-XN1376A became shorter, and the number of osmiophilic granules and granum decreased (**Figure 6H**), indicating that the photosynthetic activity and lipid accumulation ability of chloroplasts has decreased. From the binucleate stage, there was obvious difference in chloroplasts morphology between CMS-XN1376A and XN1376B (**Figures 6D, I**). In XN1376B, the chloroplast is further lengthened, and the photosynthetic capacity is also enhanced. The number of osmiophilic granules continues to increase, but their volume does not increase. The large quantity of accumulated lipids has resulted in the blurring of the thylakoid structure (**Figure 6D**). In contrast, some of the chloroplasts of CMS-XN1376A begins to degrade and become partly irregular (semi-circular shape), osmiophilic granules are significantly reduced, less lipids are accumulated, and thus thylakoid structure can be clearly observed (**Figure 6I**). At the trinucleate stage, the osmiophilic granules of XN1376B were augmented but did not increase, and starch granules were accumulated. Furthermore, the lamellar structure of the thylakoid can still be clearly observed (**Figure 6E**). By contrast, in CMS-XN1376A, the chloroplast number decreased rapidly and the structure was significantly damaged (round shape). The osmiophilic granules and thylakoids had completely disintegrated, and a large number of vacuoles appeared (**Figure 6J**).

**Figure 7:**
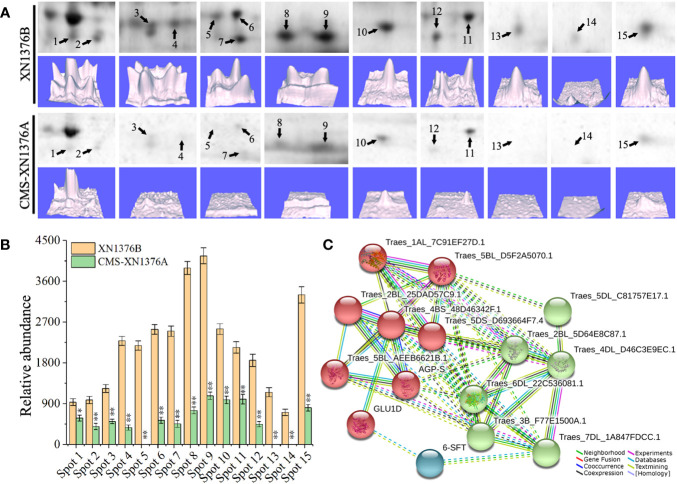
Comparison of 15 DEPs of chloroplast proteomes from the XN1376B and CMS-XN1376A. **(A)** 15 DEPs of chloroplast at the early uninucleate, and the corresponding three-dimensional images (lower panels) of expression using PDQuest software. **(B)** Abundance profiles of 15 DEPs (Spot 1-15, **Table S2**). **(C)** Analysis of protein interaction networks using the STRING system. All the homologous proteins are listed in **Table S4**.

To investigate the changes of the chloroplast proteome in S-type cytoplasmic male sterility, chloroplast protein isolated from three independent samples were separated by 2-DE (**Figure S2**). When comparing the XN1376B and CMS-XN1376A plants at the early uninucleate stage, statistical analysis of 2-DE gels showed that 15 protein spots (1-15) had at least a 1.5-fold (Student’s t-test, *p* < 0.05) change in abundance (**Figure 7A**; **Table S2**). In total, all of the DEPs were down-regulated in the CMS-XN1376A. Of these, 12 spots (spots 2-4, 6-12, and 15) had a >2.0-fold change in abundance (*p* < 0.05), and spot 4 was down-regulated over 6.0-fold change, spot 1 showed a >1.5-fold change (**Figure 7B**; **Table S2**). Meanwhile, the expression patterns of the DEPs were also different, three spots (spots 5, 13, and 14) were detected only in the chloroplast of MF-XN1376B but absent in the CMS-XN1376A, spot 9 had the highest abundance compared with the other DEPs (**Figure 7B**; **Table S2**). All the proteins were successfully identified after subjected to MALDI-TOF/TOF MS analysis, a total of seven Calvin cycle-related proteins constituted the largest proportion and the proportion was 47% (spots 3, 7, 8, 10, 13-15), and the other proteins were related to sucrose transport and metabolism (**Table S3**).

To better understand the interaction of the identified proteins, we performed Gene Ontology (GO) analysis using the online STRING 11 software (**Figure 7C**; **Tables S4**; **S5**). All 15 DEPs were blasted against the *Triticum aestivum* proteins database (**Table S4**). According to STRING analysis, the DEPs were primarily involved in the Carbohydrate metabolic process (GO:0005975, FDR=2.11e-9), Carbohydrate biosynthetic process (GO:0016051, FDR=2.49e-7), Reductive pentose-phosphate cycle (GO:0019253, FDR=2.78e-5), Cellular metabolic process (GO:0044237, FDR=4.74e-5), Phosphorylation (GO:0016310, FDR=5.49e-5), Generation of precursor metabolites and energy (GO:0006091, FDR=2.00e-4), and Cellular biosynthetic process (GO:0044249, FDR=9.10e-4; **Figure 7C**; **Table S5**). More significantly, the main protein clusters were found to be involved in the metabolism of carbohydrates, suggesting that the sugar metabolism of S-type cytoplasmic male sterility was severely inhibited.

These findings indicated that S-type cytoplasmic male sterility anthers have abnormal proteome composition, severely damaged chloroplast ultrastructure, and reduced chloroplast numbers. Due to these modifications, chloroplasts no longer perform essential tasks like self-photosynthesis and exogenous nutrient conversion, which results in a lack of nutrients for the development of microspores.

### Sugar accumulation and sugar-metabolizing enzymes activities

**Figure 8:**
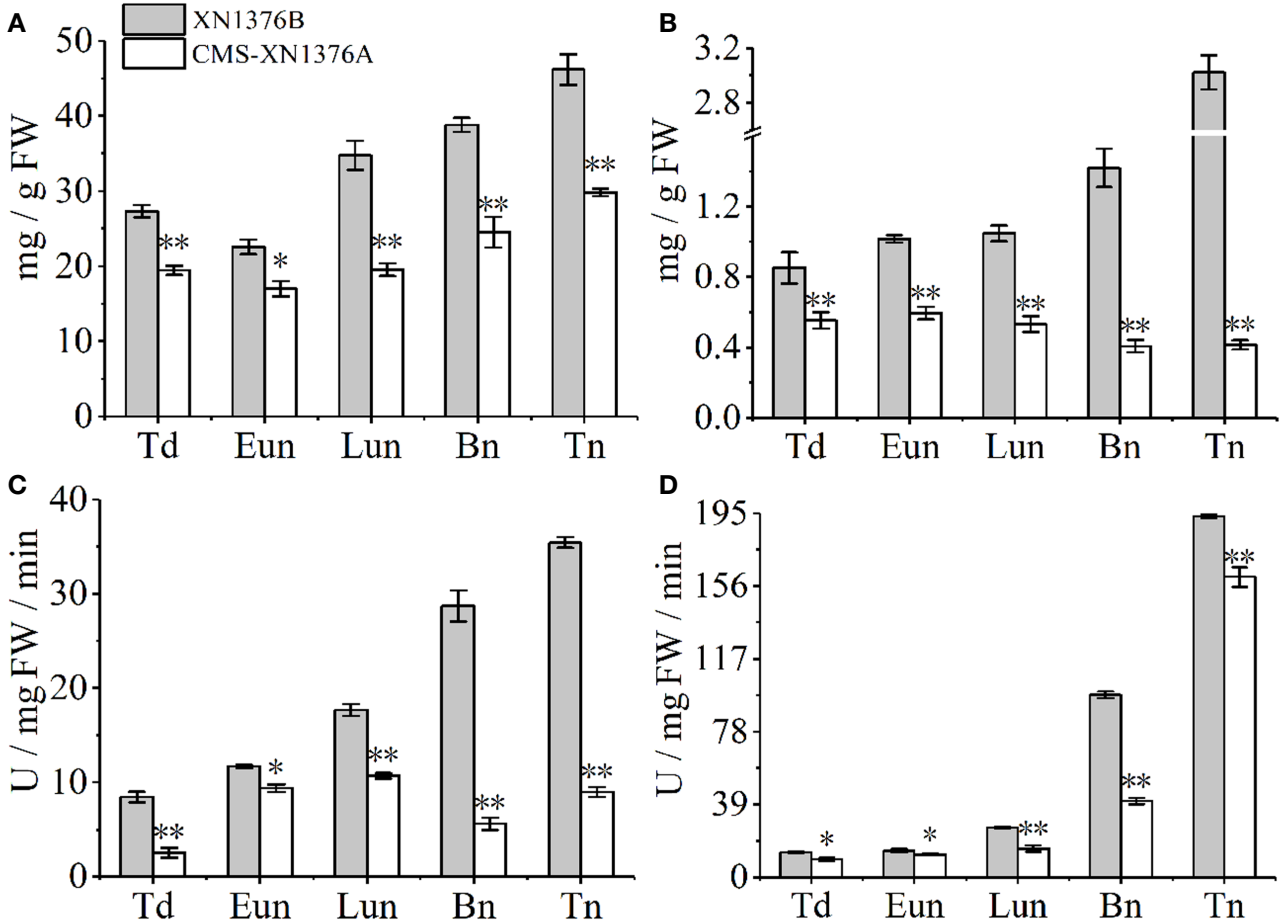
Sugar and starch contents measurement, and sugar-metabolizing enzyme activity analysis. **(A)** Sugars content. **(B)** Starch content. **(C)** Cell wall invertase (CWI) activity. **(D)** Vacuolar invertase (VIN) activity. Bn, binucleate stage; Eun, early uninucleate stage; Lun, later uninucleate stage; Td, tetrad stage; Tn, trinucleate stage. Data are means ± SD of three independent experiments (biological replicates). The significant of differences was assessed by Student’s t-test (*P < 0.05, **P < 0.01).

To further investigate the dynamic changes of sugar metabolism during anther development, sugar and starch contents and sugar-metabolizing enzyme activities were measured (**Figure 8**).

Compared with XN1376B anthers, the contents of sugars were significantly changed in CMS-XN1376A anthers from the tetrad stage to the trinucleate stage, which was repressed by 1.4-fold (*p* =0.00217) at the tetrad stage, 1.3-fold (*p*=0.01093) at the early uninucleate stage, 1.8-fold (*p*=0.00107) at the later uninucleate stage, 1.6-fold (*p*=0.00672) at the binucleate stage, and 1.5-fold (*p*=0.000321707) at the trinucleate stage, respectively (**Figure 8A**). Meanwhile, the starch contents decreased in anthers of CMS-XN1376A during anther development (decreased by 1.5- to 7.3-fold) and reached a minimum level at the trinucleate stage compared with the XN1376B anthers (**Figure 8B**).

Invertase, which can be further divided into CWI and VIN according to their subcellular location, has recently been proven to catalyze the irreversible decomposition of sucrose into glucose and fructose (Ruan, 2014; Yang et al., 2017a). These enzyme activities were then analyzed using spectrophotometric assays (**Figures 8C, D**). the activity of CWI was significantly decreased at the tetrad stage (3.3-fold, *p*=0.00246), early uninucleate stage (1.2-fold, *p*=0.01053), later uninucleate stage (1.6-fold, *p*=0.00062), binucleate stage (5.1-fold, *p*=0.00026), and trinucleate stage (3.9-fold, *p*=0.00014) of CMS-XN1376A anthers (**Figure 8C**). Also, from the tetrad to the trinucleate stage, compared with XN1376B anthers, the activity of VIN was lower (16-139.3%) in CMS-XN1376A anthers than that in XN1376B anthers (**Figure 8D**).

These findings demonstrated that the availability of carbohydrates was insufficient during anther development, which triggered microspore abortion.

### Expression levels of sugar metabolism-related genes

**Figure 9:**
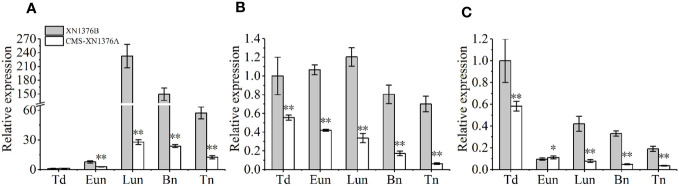
qPCR expression levels of TaCWI1 **(A)**, TaIVR5 **(B)**, and TaSUT1A **(C)**. Bn, binucleate stage; Eun, early uninucleate stage; Lun, later uninucleate stage; Td, tetrad stage; Tn, trinucleate stage. Data are means ± SD of three independent experiments (biological replicates). The significant of differences was assessed by Student’s t-test (*P < 0.05, **P < 0.01).

To further check the expression levels of genes related to sugar metabolism and transport in CMS-XN1376A and XN1376B anthers at the five anther development stages, three genes were chose to perform qPCR, which include sugar metabolism related genes (*TaCWI1* and *TaIVR5*, **Figures 9A, B**) and one sugar transport related gene (*TaSUT1A*, **Figure 9C**). Compared with XN1376B plants, the expression levels of *TaCWI1*, *TaIVR5*, and *TaSUT1A* were significantly changed in CMS-XN1376A anthers during anther development. Of them, *TaCWI1* was significantly decreased by 2.8- to 8.4-fold (*p <*0.05) in CMS-XN1376A anthers from the early uninucleate to the trinucleate stage (**Figure 9A**). *TaIVR5* was significantly decreased by 0.9- to 6.9-fold (*p <*0.05) from the tetrad to the trinucleate stage (**Figure 9B**). Similarly, *TaSUT1A* was significantly decreased by 1.8- to 11.2-fold (*p <*0.05) during anther development (**Figure 9C**). The results were essentially consistent with the abnormal sugar metabolism in the anther of CMS-XN1376A.

## Discussion

### Morphological characteristics of S-type cytoplasmic male sterility anthers

In wheat plants, anther development follows a tightly controlled sequence of events, including proper tapetum degradation and adequate nutritional supply (Bedinger, 1992; Jung et al., 2005; Xu et al., 2014; Wang et al., 2015). Numerous studies have shown that there are differences in anther abortion period and characteristics among different plants, and more than 50% of pollen abortion occurs at the tetrad stage (Wang et al., 2015; Liu et al., 2018b). However, in CMS-XN1376A, anther abortion was initially observed at the early uninucleate stage, and completely anther abortion was observed at the trinucleate stage. Meanwhile, most studies have proven that defective tapetum development always leads to abnormal pollen development, including premature and delayed degeneration of the tapetum (Wang et al., 2015; Liu et al., 2018a). Similarly, in this study, we also observed that the tapetum cells of CMS-XN1376A became abnormal at the tetrad stage and completely degraded at the binucleate stage, which showed physiological characteristics of premature degradation.

### Chloroplast dysfunction of S-type cytoplasmic male sterility anthers

**Figure 10:**
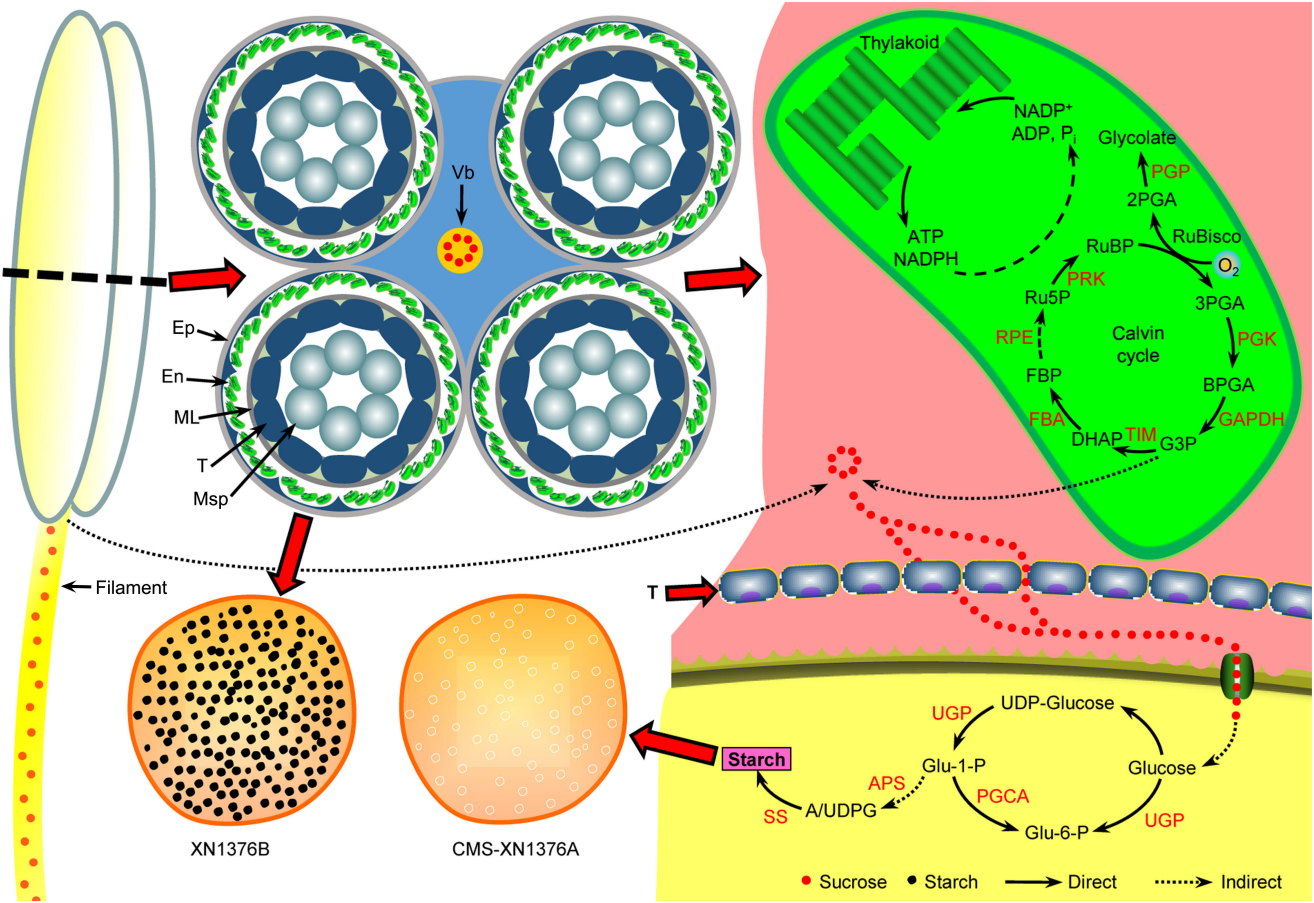
Anther abortion carbohydrate metabolism pathway in S-type cytoplasmic male sterility. In the anthers of S-CMS: due to premature degradation of tapetum, the sucrose supply for the normal development of microspores was interrupted by unloading into anthers through the apoplastic or symplastic pathway. Meanwhile, with the dysfunction of chloroplasts in the epidermal cells of the anthers, the production of sucrose in the anthers is further reduced. Ultimately, microspore abortion occurs due to insufficient nutrient supply. APS, glucose-1-phosphate adenylyltransferase small subunit; Ep, epidermis; En, endothecium; FBA, fructose-bisphosphate aldolase; ML, middle layer; Msp, microspore; PGCA, phosphoglucomutase; PGK, phosphoglycerate kinase; PGP, phosphoglycolate phosphatase; PRK, phosphoribulokinase; RPE, ribulose-5-phosphate-3-epimerase; SS, starch synthase; T, tapetum; TIM, triosephosphate isomerase; UGP, UTP-glucose-1-phosphate uridylyltransferase; Vb, vascular bundle.

In higher plants, some studies have confirmed that anthers contained a large amount of chloroplasts, which were mainly present in endothecium cells (Mamun et al., 2005; Zhu et al., 2020). These chloroplasts play a major role in starch turnover and energy conversion during anther development (Zhu et al., 2020). In this study, chloroplast was only found in the anther endothecium of wheat in this study, and its ultrastructure was significantly damaged in CMS-XN1376A, the layer structure became loose, swelled, and vacuolized, causing chloroplast dysfunction. Additionally, the chloroplasts gradually disintegrated with the development of anthers, and their number decreased rapidly. Protein is tightly connected to the life activities of eukaryotes, and specific proteins directly determine the morphological structure and physiological functions of cells (Song et al., 2015; Li et al., 2019). In this study, we identified 15 DEPs in the chloroplast of CMS-XN1376A; of them, seven proteins (47% in total) were found to be involved in the Calvin cycle (**Figure 10**), including Phosphoglycerate kinase (PGK, spot 3), Phosphoribulokinase (PRK, spot 7), Glyceraldehyde-3-phosphate dehydrogenase GAPA1 (GAPDH, spot 8), Fructose-bisphosphate aldolase 2 (FBA, spot 10), Triosephosphate isomerase (TIM, spot 13), Phosphoglycolate phosphatase 1A (PGP, spot 14), Ribulose-5-phosphate-3-epimerase (RPE, spot 15), and covered a wide range of carboxylation, and reduction and regeneration processes of the Calvin cycle. As we know, the cycle occurs in the chloroplasts of plants, and are important for the fixation of carbon dioxide and the formation of a 6-carbon sugar (Singh and Malhotra, 2000). PGP is known to be involved in the subpathway that synthesizes glycolate from 2-phosphoglycolate produced by the RuBisCO oxygenation reaction (Ruan, 2014; Li et al., 2023). In the reduction processes, 3PGA is phosphorylated under the catalysis of PGK to generate BPGA and then reduced to glyceraldehyde G3P by GAPDH. Meanwhile, GAPDH plays a central role in the Calvin cycle that assimilates CO_2_ (Yang et al., 2017a; Li et al., 2023). Subsequently, G3P is regenerated into RUBP under the action of a series of enzymes, including TIM, FBA, RPE, and PRK (Leegood, 1993; Agarwal et al., 2009). In the present study, the expression of proteins involved in the Calvin cycle was suppressed, which inhibited photosynthesis, caused the loss of carbon fixation, and ultimately reduced the production of sugar and biomass.

### Carbohydrate metabolism of S-type cytoplasmic male sterility anthers

Plants mainly use photosynthesis to fix and store light energy in the form of sugar molecules, which are essential for plant energy circulation and survival (Singh and Malhotra, 2000). Meanwhile, sucrose is the end product of photosynthesis and the primary carbohydrate transported in most plants (Leegood, 1993). Moreover, anther development requires lots of nutrients from energy organs, and studies have shown that the chloroplast in the endothecium of anthers has starch turnover ability in the early developmental stages and photosynthesis ability in the later stages, which can provide necessary nutrients for pollen development (Clément and Audran, 1999; Castro and Clément, 2007; Oliver et al., 2010; Zhu et al., 2020). Once the supply of nutrients is insufficient, pollen fertility will decrease, resulting in anther abortion (Datta et al., 2002; Castro and Clément, 2007). In this study, in the sterile line anthers, the chloroplast structure is damaged and the Calvin cycle is inhibited, resulting in decreased sucrose content in the chloroplast of anthers.

In wheat, anthers are organs with the strongest reservoirs in flowers, and their development requires nutrients (mainly sucrose) stored in source organs (Koch, 1996; Smeekens et al., 2010; Zhu et al., 2015). As shown in **Figure 10**, sucrose from leaves (transported by filaments) and anthers is unloaded to the developing anthers through the tapetum cells either apoplasmically or symplasmically (Zhu et al., 2015; Yang et al., 2017a). Meanwhile, there is a certain cooperative mode between the tapetum and chloroplasts in the endothecium of anthers: lipid metabolism and its products in the tapetum promote the formation of chloroplast membrane in the endothecium, ensuring the normal structure and function of chloroplasts; the starch turnover and sugar metabolism pathway in the chloroplasts of the endothecium, as well as their products, promote the development of the anther tapetum (especially under night or dark conditions); the tapetum and chloroplast of endothecium work together to provide lipid, carbohydrate substances and energy for the final formation of mature pollen grains (Zhu et al., 2020). Additionally, sucrose imported into anther via plasmodesmata or taken up by SUT may be degraded by cytoplasmic invertase and source synthase (Yang et al., 2017a). However, studies have confirmed that the plasmodesmata between the anther tapetum and other cell layers disappeared from the meiotic phase, and sucrose needs to be transported from tapetum to microspores through the alternative appoplastic sugar supply pathway, which involves invertase (CWI and VIN) and SUT (Ruan, 2014). Of them, CWI could hydrolyze sucrose into UDP-glucose and fructose before being taken up into the cytoplasm (Castro and Clément, 2007). VIN can hydrolyze cytosolic sucrose to hexose (Wolswinkel and Ammerlaan, 1983; Ruan, 2014). In the present study, the results showed that the activities of CWI and VIN were repressed in CMS-XN1376A plants from the tetrad stage to the trinucleate stage (**Figure 8**). And qPCR analysis indicated that two invertase-related genes (*CWI* and *IVR5)* had decreased expression level in CMS-XN1376A (**Figure 9**). Furthermore, the sucrose transport gene *TaSUT1A* was down-regulated in CMS-XN1376A plants at the five stages (**Figure 9C**). These changes imply that sucrose degradation and transport are subjected to different degrees of inhibition. Recent studies showed that sugars entering into microspores are catalyzed by starch synthase to synthesize starch granules (Yang et al., 2017a; Wang et al., 2019). Moreover, after measured the total soluble sugar and starch contents, we found that they were significantly decreased in CMS-XN1376. This indicated that the microspores of male sterility had fewer carbohydrates during their development. Additionally, proteins (spots 1, 2, 5, 6, 9, 11, and 12) involved in sucrose degradation and starch synthesis during pollen abortion have been documented by Wang et al. (2019). Based on previous reports and findings, a tentative model of carbohydrate metabolism pathway for anther abortion in S-type cytoplasmic male sterility was proposed, as summarized in **Figure 10**.

In different plant tissues, the distribution and quantity of chloroplasts are different, and they are mainly concentrated in mesophyll cells of leaves (Kirchhoff, 2019). The photosynthetic products produced and the energy metabolism mediated by chloroplasts are crucial for the development of anthers, especially in the early developmental stages (where the chloroplasts of anthers lack photosynthetic capacity; Zhu et al., 2020). Therefore, in the early development stages of S-type cytoplasmic male sterility anther, whether the morphological structure, protein composition, and photosynthetic products of chloroplasts in the leaves are normal, and whether the starch turnover in the early stages of anthers comes from the photosynthetic products of leaves, all these are the focus of our future research.

## Conclusions

In this study, we investigated the cytological changes and carbohydrate metabolism dynamics of the S-type cytoplasmic male sterility in wheat, where we found that anthers of S-CMS undergo premature tapetal degradation, abnormal microspore development, and gradual nutritional shortage. More importantly, aberrant chloroplast ultrastructure and chloroplast proteins reduced chlorophyll synthesis, photosynthetic capacity, and carbon dioxide fixation. We suggested that the metabolism and transportation of sugar in anthers of S-CMS are changed, thereby the resource supply of microspores are reduced, which ultimately leads to weak male function in S-type cytoplasmic male sterility in wheat.

## Data availability statement

The original contributions presented in the study are included in the article/**Supplementary Material**, further inquiries can be directed to the corresponding author/s.

## Author contributions

SW: Conceptualization, Writing – original draft, Writing – review & editing. SG: Conceptualization, Writing – original draft. FD: Conceptualization, Writing – original draft, Writing – review & editing. BD: Conceptualization, Writing – review & editing. CW: Data curation, Writing – review & editing. MR: Data curation, Software, Writing – review & editing. CM: Data curation, Software, Writing – review & editing. YX: Methodology, Writing – review & editing. DZ: Methodology, Writing – review & editing. YL: Writing – review & editing. ZZ: Writing – review & editing. ZF: Writing – review & editing. GZ: Writing – review & editing. YZ: Conceptualization, Writing – original draft, Writing – review & editing.

## Supplementary material

Supplementary Figure 1 2-DE electrophoresis gels of chloroplast proteomes in the XN1376B and CMS-XN1376A wheat plants at the early uninucleate stage. Chloroplast proteins (200 μg) were loaded on IPG gel strips (17-cm; pH 4-7), then followed by 12% SDS-PAGE. Proteins were visualized using silver staining. Numbered spots represent the identifications detailed in **Table S2**.
