# Supplementary figures and images for: Carbohydrate metabolism and cytology of S-type cytoplasmic male sterility in wheat
Source: Front Plant Sci. 2023 Oct 16;14:1255670. doi: 10.3389/fpls.2023.1255670 (PMC10614052; doi:10.3389/fpls.2023.1255670)

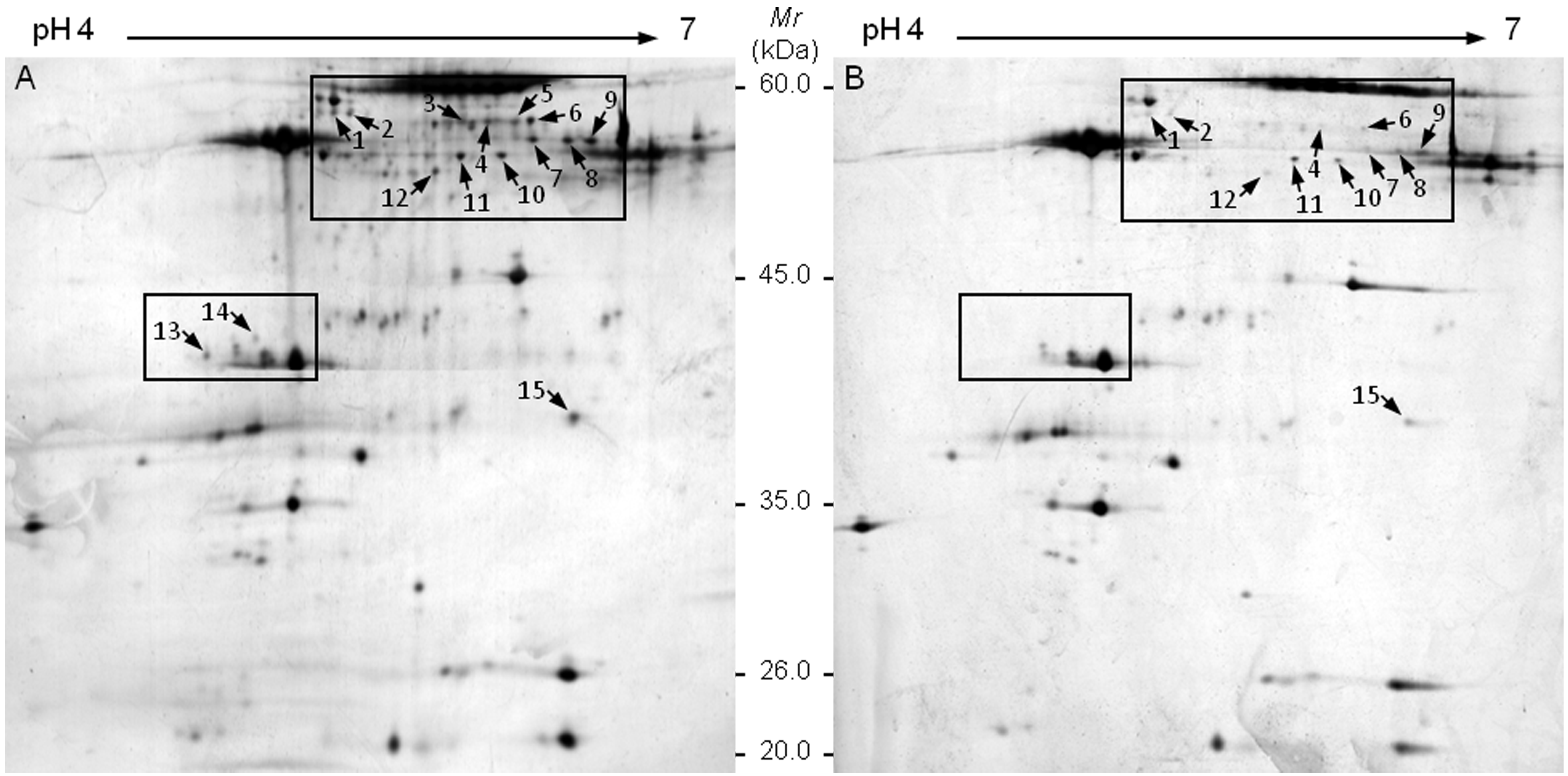

Supplement: Supplementary Figure 1 — 2-DE electrophoresis gels of chloroplast proteomes in the XN1376B and CMS-XN1376A wheat plants at the early uninucleate stage. Chloroplast proteins (200 μg) were loaded on IPG gel strips (17-cm; pH 4-7), then followed by 12% SDS-PAGE. Proteins were visualized using silver staining. Numbered spots represent the identifications detailed in Table S2 . [file Image_1.tif]
